# Lutein-Loaded, Biotin-Decorated Polymeric Nanoparticles Enhance Lutein Uptake in Retinal Cells

**DOI:** 10.3390/pharmaceutics12090798

**Published:** 2020-08-24

**Authors:** Pradeep Kumar Bolla, Vrinda Gote, Mahima Singh, Manan Patel, Bradley A. Clark, Jwala Renukuntla

**Affiliations:** 1 Department of Basic Pharmaceutical Sciences, Fred Wilson School of Pharmacy, High Point University, High Point, NC 27262, USA; bollaniper@gmail.com (P.K.B.); bclark@highpoint.edu (B.A.C.); 2 Division of Pharmacology and Pharmaceutical Sciences, School of Pharmacy, University of Missouri, 2464 Charlotte Street, Kansas City, MO 64108, USA; vrindagote@mail.umkc.edu; 3 Department of Pharmaceutical Sciences, University of the Sciences in Philadelphia, Philadelphia, PA 19104, USA; msingh@mail.usciences.edu (M.S.); mpatel@biolinkonline.com (M.P.)

**Keywords:** lutein, PLGA, PLGA–PEG–biotin, ARPE-19, retina, macular edema, age-related macular degeneration, biotin-decorated nanoparticles, polymeric nanoparticles, targeted therapy

## Abstract

Age related macular degeneration (AMD) is one of the leading causes of visual loss and is responsible for approximately 9% of global blindness. It is a progressive eye disorder seen in elderly people (>65 years) mainly affecting the macula. Lutein, a carotenoid, is an antioxidant, and has shown neuroprotective properties in the retina. However, lutein has poor bioavailability owing to poor aqueous solubility. Drug delivery to the posterior segment of the eye is challenging due to the blood–retina barrier. Retinal pigment epithelium (RPE) expresses the sodium-dependent multivitamin transporter (SMVT) transport system which selectively uptakes biotin by active transport. In this study, we aimed to enhance lutein uptake into retinal cells using PLGA–PEG–biotin nanoparticles. Lutein loaded polymeric nanoparticles were prepared using O/W solvent-evaporation method. Particle size and zeta potential (ZP) were determined using Malvern Zetasizer. Other characterizations included differential scanning calorimetry, FTIR, and in-vitro release studies. In-vitro uptake and cytotoxicity studies were conducted in ARPE-19 cells using flow cytometry and confocal microscopy. Lutein was successfully encapsulated into PLGA and PLGA–PEG–biotin nanoparticles (<250 nm) with uniform size distribution and high ZP. The entrapment efficiency of lutein was ≈56% and ≈75% for lutein-loaded PLGA and PLGA–PEG–biotin nanoparticles, respectively. FTIR and DSC confirmed encapsulation of lutein into nanoparticles. Cellular uptake studies in ARPE-19 cells confirmed a higher uptake of lutein with PLGA–PEG–biotin nanoparticles compared to PLGA nanoparticles and lutein alone. In vitro cytotoxicity results confirmed that the nanoparticles were safe, effective, and non-toxic. Findings from this study suggest that lutein-loaded PLGA–PEG–biotin nanoparticles can be potentially used for treatment of AMD for higher lutein uptake.

## 1. Introduction

The eye is considered as one of the most sophisticated sensory organs of the human body due to its intricate anatomical structure. Anatomically, the eye can be broadly classified into two segments; i.e., (a) the anterior segment comprising cornea, aqueous humor, conjunctiva, ciliary body, iris, and lens, and (b) the posterior segment consisting of sclera, choroid, Bruch’s membrane, retinal pigment epithelium, retina, optic nerve, and vitreous humor [1,2]. All the components of the anterior and posterior segments co-ordinate functionally and enable vision formation [3]. Alterations in the arrangement of these structures due to several factors such as aging, infection, inflammation, exposure to UV light, injury, air pollution, and over/under secretion of ocular fluids result in ocular diseases. Based on the localization, the diseases can be categorized into diseases affecting the anterior segment (ocular pain and inflammation, allergic conjunctivitis, blepharitis, keratitis, sty, anterior uveitis, and glaucoma) [4] and posterior segment (macular edema (cystic and diabetic macular edema), retinitis, age-related macular degeneration (AMD), proliferative vitreoretinopathy, diabetic retinopathy, choroidal neovascularization, and others) [3]. AMD is one of the leading causes of visual loss and is responsible for approximately 9% of global blindness [5]. It is a progressive eye disorder common in the elderly population (>65 years) mainly affecting the macula (central region of the retina) which is responsible for vision [6]. In 2012, it was estimated that 50 million people worldwide and 10 million people in the US suffered from AMD [6]. Till now, there was no clear understanding of the pathophysiology of AMD; however, it is a complicated disorder involving several risk factors which include smoking, UV light exposure, inflammation, and genetic factors [6,7,8]. The early stage of AMD is characterized by the deposition of yellowish deposits, known as soft drusen accumulations, in the retinal pigment epithelium and Bruch’s membrane. The later stage of the disease is associated with loss of vision due to atrophy of photoreceptors and retinal pigment epithelium, retinal scarring, and detachment of retina [6,8]. If untreated, AMD is the leading cause of vision loss in 45% of all visual disability cases in the US alone [7]. Currently, there is no cure for AMD, and very few treatments such as anti-vascular endothelial growth factor (VEGF) have been proven to slow the progression of AMD. In addition, it is hypothesized that antioxidants and anti-inflammatory agents such as carotenoids (lutein, zeaxanthin, α-carotene, β-carotene, lycopene, and β-cryptoxanthin) protect against AMD by absorbing UV light, reducing oxidative stress, and stabilizing cell membranes [9].

Lutein is a dihydroxy xanthophyll carotenoid (β,ε-carotene-3,3′-diol) and is ubiquitously available from a variety of green leafy vegetables, fruits, flowers, egg yolk, etc. [10,11]. Since humans/animals cannot synthesize lutein, it must be obtained from the diet. It has been reported in the literature that lutein intake has improved the visual acuity and prevented the progression on AMD [12]. In 2016, Allison et al. showed that lutein was selectively taken up by the retinal pigment epithelial cells and showed protection against oxidative stress induced damage [13,14]. In addition, a recently completed clinical study has also demonstrated the protective effects of lutein in early stage AMD. Thus, it is hypothesized that lutein supplementation could halt the progression of AMD by reducing the oxidative stress in the retina caused by hypoxia and intense exposure to UV light [6]. Unfortunately, lutein has poor bioavailability due to high lipophilicity (log P 7.9) and poor aqueous solubility. [15,16].

Management of ocular/ophthalmic diseases is mainly achieved by using conventional topical products such as ophthalmic solutions, suspensions, and ointments [17]. However, the bioavailability of drugs administered by these conventional drug delivery systems is very low, ranging from 1% to 5% for hydrophobic drugs and <0.5% for hydrophilic drugs [17]. Drug delivery to the posterior part of the eye is challenging as the diseases affecting posterior segment require long-term delivery at a higher dose to the targeted tissues such as retina, choroid and Bruch’s membrane. Moreover, treatment strategies such as oral, intraocular, and periocular routes have limited success due to the presence of static barriers such as sclera, retinal pigment epithelium (RPE), and multidrug resistance efflux pumps [3,18]. Various approaches have been explored in improving the ocular bioavailability, which include traditional formulation improvements, use of prodrugs, and carrier mediated drug transport. Formulation improvements include development of novel formulations such as suspensions, ointments, gels, nanoparticles, solutions, microemulsions, niosomes, liposomes, micelles, and others [1,2,17]. Transport of drugs, ions, and nutrients into and out of the ocular cell occurs mainly through transporters, receptors, and transmembrane proteins [19,20,21]. Transmembrane transporters/receptors are also involved in cellular processes such as absorption, distribution, and elimination of xenobiotics and nutrients [21,22]. Several transporters/receptors have been identified in the eye, which include glucose transporters, peptide transporters, amino acid transporters, nucleoside/nucleobase transporters, vitamin transporters, and nutrient receptors [21,22]. Various vitamin transporters have been characterized on the retinal epithelium which include folate, biotin, and ascorbic acid [20,22]. Transporter-mediated drug delivery can be achieved by conjugation of drug to a specific substrate/nutrient such as folic acid or biotin. The nutrient will be recognized by the transporter proteins and conjugated drug is translocated across the cell membrane thus increasing permeability [22].

Biotin (vitamin B7) is an essential water-soluble vitamin useful for cell growth, function, and development. It is a co-factor for several carboxylase enzymes which catalyze multiple metabolic reactions and acts as a regulator in cell signaling pathways and gene expression. Biotin cannot be synthesized in mammalian cells and therefore must be obtained from exogenous sources. Biotin transport in cells is mediated through sodium-dependent multivitamin transporter (SMVT) or biotin transporter. Biotin transporter is a high-affinity transporter involved in transport of biotin, whereas SMVT is a low-affinity transporter involved in the transport of biotin, pantothenic acid, and lipoic acid. It has been reported in the literature that SMVT is abundantly expressed in the blood–retina barrier and retinal cells (D407 cells). Thus, it is known that biotinylated prodrugs and polymeric nanoparticles utilize the SMVT and biotin transporters for enhanced permeability of drugs [19,23]. Moreover, scientists have reported that biotin-decorated polymeric nanoparticles have enhanced the uptake of poorly soluble drugs such as doxorubicin [24], SN-38 [25], and 15,16-dihydrotanshinone [26]. Therefore, we hypothesize that lutein-loaded PLGA–PEG–biotin polymeric nanoparticles can enhance the uptake of lutein into the retinal cells through SMVT transport system.

Polymeric nanoparticles are known to improve the bioavailability of drugs with poor biopharmaceutical properties [27,28]. Furthermore, polymeric nanoparticles have advantages, including biocompatibility, enhanced stability, sustained release, and improved efficacy [28,29,30,31,32]. Polymeric nanoparticles are prepared using biodegradable polymers such as poly(lactide co-glycolide) (PLGA), gelatin, chitosan, albumin, alginate polycaprolactone, polyglycolides, poly (methyl methacrylate), and polyethylene glycol (PEG) [28,33]. Previously, lutein was encapsulated into several nanocarriers, such as PLGA nanoparticles, liposomes, nanoemulsions, nanocrystals, lipid nanocapsules, and nanodispersions [34,35,36,37,38,39,40]. In the present study we aimed to prepare and characterize lutein-loaded polymeric nanoparticles (PLGA and PLGA–PEG–biotin) and evaluate their enhanced uptake in retinal cells.

## 2. Materials and Methods

### 2.1. Materials

Lutein (90%) was purchased from Acros Organics (Fair Lawn, NJ, USA). Dimethyl sulfoxide (DMSO), methanol (HPLC grade), tetrahydrofuran (HPLC grade), dichloromethane, sodium chloride, disodium hydrogen phosphate, potassium dihydrogen phosphate, MTT (3-(4,5-dimethylthiazol-2-yl)-2,5-diphenyltetrazolium bromide), and ammonium chloride (NH_4_Cl) were purchased from Fisher Scientific (Fair Lawn, NJ, USA). Sodium dodecyl sulphate (SDS) and polyvinyl alcohol (PVA: MW: 30,000–70,000) were procured from Sigma Aldrich (St. Louis, MO, USA). PLGA (50:50; Mw: 10,000 Da) and PLGA–PEG–biotin (50:50; Mw: 10,000 Da–2000 Da) were purchased from Akina PolySciTech, Inc, West Lafayette, IN, USA. Dulbecco’s modified Eagle medium (DMEM, Gibco’s), Dulbecco’s phosphate-buffered saline (DPBS), Triton-X, and trypsin (TrypLE, Gibco) were purchased from Thermo Fisher Scientific (Fair Lawn, NJ, USA). Cellulose ester dialysis tubing (Biotech grade; Mw: 300 kDa) was procured from Spectrum Laboratories, Inc (Gardena, CA, USA). Fluorescein isothiocyanate (FITC) and 4′,6-diamidino-2-Phenylindole (DAPI) were purchased from Invitrogen, Labelling and Detection, Molecular Probes, ThermoFisher Scientific, (Fair Lawn, NJ, USA). Human retinal pigment epithelial cell line (ARPE-19) was purchased from American Type Culture Collection (ATCC, Manassas, VA, USA).

### 2.2. Methods

#### 2.2.1. Preparation of Lutein-Loaded Polymeric Nanoparticles

**Figure 1:**
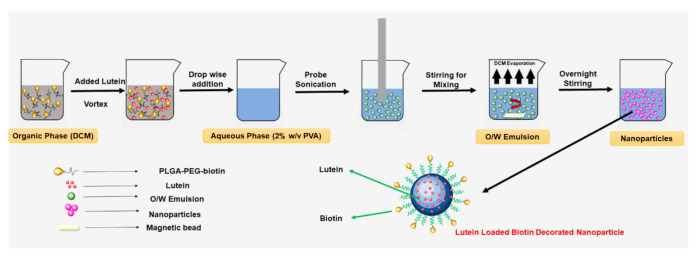
Schematic representation of oil in water emulsion-solvent evaporation method for preparation of lutein loaded PLGA-PEG-biotin nanoparticles.

Lutein-loaded polymeric nanoparticles were prepared using the oil-in-water (O/W) emulsion solvent evaporation method reported earlier with slight modifications (Figure 1) [41,42]. In brief, 100 mg of polymer (PLGA or PLGA–PEG–biotin) was dissolved in 5 mL of dichloromethane and lutein (20 mg) was dissolved in 2 mL dichloromethane separately. The polymer and lutein solutions were mixed to form a homogenous organic phase. The organic phase was sonicated in a bath sonicator for 5 min followed by slow addition to an aqueous solution of 2% PVA (20 mL) under continuous stirring on a magnetic stirrer. The resultant mixture was sonicated at 30% amplitude for 5 min using a probe sonicator (Fisher Scientific^™^ Model 505 Sonic Dismembrator) to obtain an emulsion. The sonication step was performed in an icebath to prevent overheating of the emulsion. After sonication, the emulsion was stirred gently at room temperature overnight until complete evaporation of dichloromethane. Un-entrapped lutein and PVA residue were removed from the emulsion by washing three times with deionized water using Hitachi ultracentrifuge at 22,000× *g* for 1 h. Finally, the nanoparticles formed were lyophilized using laboratory freeze dryer (Harvestright, Salt Lake City, UT, USA) for 24 h.

#### 2.2.2. Determination of Size, Polydispersity Index, and Zeta Potential

The particle size, polydispersity index (PDI), and zeta-potential (ZP) of lutein-loaded PLGA and PLGA–PEG–biotin nanoparticles were measured using the dynamic light scattering (DLS) technique. The nanoparticles (200 µL) were dispersed in 10 mL of double distilled de-ionized water and measurements were determined using Malvern Zetasizer Nano ZS90 (Malvern Instruments, Malvern, UK) at 25 °C. All the measurements were performed in triplicate (n = 3).

#### 2.2.3. Lutein Quantification Using HPLC

The amount of lutein in the samples was quantified using Waters Alliance e2695 HPLC (Waters Corporation, Milford, MA, USA) equipped with 2996 photodiode array (PDA) detector and Empower 2.0 software. The analysis was performed using reverse-phase Waters^®^ C-18 column (5 µm; 250 mm × 4.6 mm) under isocratic conditions (flow rate of 1 mL/min at 25 °C). Mobile phase was a mixture (90:10) of methanol and tetrahydrofuran. The analyte was monitored at 450 nm. Sample injection volume was 20 µL and the run time was 10 min. Retention time of lutein was 3.85 min [43]. All the samples injected were filtered through 0.45 µm membrane filter. Stock solution (1 mg/mL) of lutein was prepared in the mobile phase, and calibration standards (n = 3) ranging from (1 µg/mL to 100 µg/mL) were serially diluted in the mobile phase. Similar standard curve was also prepared by dissolving lutein in DMSO. Calibration curves were obtained by plotting peak area against the concentration of lutein. The lutein content in the samples was determined quantitatively using the linear regression equations from the calibration curves (R^2^ > 0.99). The HPLC method provided rapid and reproducible results without a significant difference in intra and inter-day analysis.

#### 2.2.4. Determination of Lutein Encapsulation Efficiency (%EE) and Drug Loading (%DL)

The encapsulation and loading of lutein into nanoparticles were determined by quantifying the lutein content in freeze dried nanoparticles using HPLC. In brief, the freeze-dried nanoparticles (10 mg) were dissolved in 10 mL DMSO and the amount of lutein was determined using HPLC. The %EE and %DL of lutein in the nanoparticles were determined using the following formulae (Equations (1) and (2)). All the measurements were performed on three different samples (n = 3).

%EE = (Amount of lutein remained in nanoparticles)/(Initial amount of lutein) × 100(1)

%DL = (Weight of lutein in nanoparticles)/(Weight of polymer used) × 100(2)

#### 2.2.5. Differential Scanning Calorimetry (DSC)

Interaction of lutein with PLGA and PLGA–PEG–biotin was confirmed using DSC technique. Calorimetric analysis was performed for lutein, PVA, polymers (PLGA and PLGA–PEG–biotin), and lutein-loaded polymeric nanoparticles using a DSC822e (Mettler Toledo, Columbus, OH, USA) instrument. Samples (3–11 mg) were accurately weighed in aluminum pans (40 µL capacity) and were hermetically sealed using a crimping device. The reference standard was an empty aluminum pan. Nitrogen was purged at a rate of 20 mL/min during the analysis. Samples were held isothermally at 25 °C for 5 min and then heated at 10 °C/min to 280 °C. All the thermograms recorded were analyzed using STARe software.

#### 2.2.6. Fourier Transform Infrared Spectroscopy (FTIR)

A JASCO-FT/IR 4600 instrument (Jasco instruments, Easton, MD, USA) using the attenuated total reflection (ATR) technique was used to record the FTIR spectra of lutein, PVA, polymers (PLGA and PLGA–PEG–biotin), and lutein-loaded polymeric nanoparticles. The sample compartment was flushed with argon prior to each run. Each sample was ground to fine powder with a KBr pellet. The scanning range was from 500–4000 cm^−1^. After measurement of the spectrum, data were analyzed and plotted. Carbon dioxide (CO_2_) and water (H_2_O) peaks were subtracted from the original spectrum to obtain the final IR spectrum.

#### 2.2.7. In-Vitro Release Studies

Drug release behavior from lutein-loaded polymeric nanoparticles was determined using the dialysis bag method (MWCO: 300 kDa) [27]. Initially, several release media were screened to determine the solubility of lutein. Phosphate-buffered saline (pH 7.4) with 0.2% *w*/*v* sodium dodecyl sulphate was chosen as the suitable release medium for release studies to maintain sink conditions (saturation solubility: 40.1 ± 8.47 µg/mL) [10]. After selecting the release medium, 1 mL lutein-loaded polymeric nanoparticles (≈1 mg lutein) were transferred to individual dialysis tubing and release medium (1 mL) was added to each tubing. Leakage was prevented by sealing the tubing tightly at both ends. Sealed dialysis tubings loaded with nanoparticles were transferred to 250 mL beakers containing 100 mL of release medium in a shaking water bath (100 rpm) (maintained at 37 ± 0.5 °C). To prevent any evaporation of release medium, beakers were tightly sealed with parafilm. At a pre-determined time-intervals (0.5, 1, 2, 3, 4, 5, 6, 7, 8, and 24 h), samples (10 mL) were collected from each beaker and replaced with 10 mL of fresh release medium. The cumulative amount of drug released from the formulations was quantified using UV spectrophotometer (6405 UV/Vis Spectrophotometer, Jenway, Staffordshire, UK) by measuring the absorbance at λ_max_ of 440 nm at different timepoints. All experiments were performed on three different samples (n = 3).

#### 2.2.8. Cell Culture Studies

##### Cell Culture

ARPE-19 cells were used to determine the in-vitro cellular uptake and cytotoxicity of lutein and lutein-loaded polymeric nanoparticles (PLGA and PLGA–PEG–biotin). ARPE-19 cells were purchased from American Type Culture Collection and stored in liquid nitrogen. The cells were cultured in DMEM/ F-12 (1:1 ratio) media containing 10% (*v*/*v*) heat-inactivated fetal bovine serum (FBS), 100 U/mL penicillin, 100 µg/mL streptomycin, 1% (*v*/*v*) MEM non-essential amino acids, and 1% sodium bicarbonate. The cells were grown in T-75 Corning flasks and incubated at 37 °C, 5% CO_2_, and 95% relative humidity and harvested at 80–90% confluency.

##### FITC Labelling

The in-vitro cellular uptake levels of lutein-loaded PLGA and PLGA–PEG–biotin nanoparticles, and lutein alone were determined by labeling (surface adsorption) the samples with FITC, which is widely used to label proteins [44], drugs [45], and polymers [46]. FITC labelling was performed according to a previously reported protocol with slight modifications [47]. In brief, 10 µg of lyophilized nanoparticles (PLGA or PLGA–PEG–biotin) were suspended in 50 mM phosphate buffered saline (1 mL) to make a 10 µg/mL of nanoparticle suspension. Separately, FITC was powdered and dissolved in DMSO (1 mg/mL) since FITC was not soluble in water. The FITC solution was added to nanoparticle suspension and incubated in the dark for 12 h at 4 °C. After incubation, 1 mL of 50 mM NH_4_Cl was added to the mixture to inactivate unreacted FITC. Further, the FITC labelled polymeric nanoparticles were subjected to dialysis to remove any unreacted FITC and NH_4_Cl. Finally, the FITC-labelled nanoparticles were filtered through a 0.22 µm nylon filter to ensure sterility. The samples were aliquoted and stored at −20 °C until further use. The sample for FITC-labelled lutein was prepared in a similar way as described above, except lutein was dissolved in DMSO (5 mg/mL) and then mixed with FITC solution.

##### In Vitro Cellular Uptake Studies Using Flow Cytometry (Fluorescence-Activated Cell Sorting (FACS))

Intracellular uptake of FITC-labelled nanoparticles and lutein alone in ARPE-19 cells was determined by incubating the cells and then evaluating time-dependent uptake using flow cytometry (FACS). ARPE-19 cells were seeded in a 12-well plate at a density of 0.5 × 10^6^ cells/ well with 1 mL of complete DMEM/F-12 media. The cells were treated with 10 µL of each sample which included control (DMSO), FITC–lutein, FITC–lutein PLGA nanoparticles, and FITC–lutein PLGA–PEG–biotin nanoparticles. Then, the treated cells were incubated for various times (3, 6, 9, and 12 h). At each time point, the media was removed from the wells and the cells were harvested by using 200 µL of trypsin (TrypLE, Gibco). This was followed by 5 min incubation and addition of serum containing DMEM/F-12 media. Further, the cells were collected in FACS tubes and centrifuged at 20,000 rpm for 5 min to obtain a cell pellet. The media was then discarded, and the cells were washed twice using 1 mL of (DPBS). The final sample was prepared in DPBS and acquired by flow cytometry to determine the mean FITC fluorescence intensity of the cells at an excitation wavelength of 490 nm. The mean FITC fluorescence intensity values obtained for all the samples (n = 3) were plotted using bar-graphs in GraphPad Prism (version 5.0) and the differences were observed.

##### In Vitro Cellular Uptake Studies Using Confocal Laser Scanning Microscopy

Intracellular distribution of FITC-labelled polymeric nanoparticles (PLGA and PLGA–PEG–biotin) in ARPE-19 cells was determined using confocal laser scanning microscopy (CLSM). FITC–lutein–PLGA–PEG–biotin nanoparticles, FITC–lutein PLGA nanoparticles and FITC–lutein were prepared using the same method described in earlier sections. The cells were seeded in an 8-chamber confocal microscopy slide precoated with collagen (Nunc Lab-Tek, Thermo Fisher Scientific, Waltham, MA, USA) with 200 µL of complete DMEM/F-12 media. This was followed by 10 µL additions of various treatment samples which included control (DMSO), FITC–lutein, FITC–lutein PLGA nanoparticles, and FITC–lutein PLGA-Peg-biotin nanoparticles into each chamber of the 8-chamber plate. Further, the treatment groups were incubated for 6 and 12 h. At each time point, the culture media was removed, and the cells were washed two times on a shaker with 300 µL of DPBS for 5 min. This was followed by fixing the cells with freshly prepared cold 4% buffered paraformaldehyde solution (200 µL) and incubating at 37 °C for 20 min. After incubation, the fixing solution was removed, and the cells were washed again using 300 µL DPBS (3 times × 5 min each). Further, the nuclei of the cells were stained with 100 µL of DAPI (working solution of 10 µg/mL) for 15 min in dark. The cells were then mounted and sealed with cover slip to prevent any evaporation of mounting media and dehydration of the cells. The ARPE-19 cell slides were stored at 4 °C before the actual analysis. A Leica Confocal Laser Scanning Microscope (Leica TCS SP5, Wetzlar, Germany) was used to analyze the cells for green fluorescence-FITC and blue fluorescence-DAPI.

##### In Vitro Cell Viability Studies (MTT Assay)

Cellular cytotoxicity of lutein PLGA–PEG–biotin nanoparticles, lutein PLGA nanoparticles and lutein alone were determined in ARPE-19 cells by using MTT (3-(4,5-dimethylthiazol-2-yl)-2,5-diphenyltetrazolium bromide) assay. MTT is a yellow tetrazole dye which is reduced to purple formazan crystals by viable or living cells. The cell viability is determined by measuring the absorbance. ARPE-19 cells were seeded at a density of 1 × 10^4^ cells in a 96-well plate. The cells were supplemented with 200 µL of DMEM/ F-12 culture media (1:1 ratio) containing 10% fetal bovine serum. The samples (lutein–PLGA–PEG–biotin nanoparticles, lutein–PLGA nanoparticles. and lutein) were prepared in serum free DMEM: F-12 media and filtered through a 0.22 µm nylon filter. A small quantity of dichloromethane (200 uL) was added to dissolve lutein in the media. Complete DMEM/F-12 from the cell lines was replaced with 100 µL solution of treatment samples prepared in serum free media. All the three treatment groups were analyzed at four concentrations which included 10, 20, and 50 µg/mL of equivalent lutein. The cells were incubated with each sample for 24 h at 5% CO_2_ and 37 °C. After incubation, the cells were washed twice with PBS. Separately, MTT reagent A and MTT reagent B were mixed in the ratio 100:1 to make a stock solution of the dye. Further, 20 µL of MTT stock solution was added to each well and incubated for 3.5 h. Finally, the absorbance of formazan solution was measured using a microplate reader (BioRad, Hercules, CA, USA) at an excitation wavelength of 485 nm. Five percent Triton-X prepared in serum free media served as the positive control and serum free media lacking any samples served as the negative control. Cell viability was calculated according to the formula (Equation (3)).

% Cell Viability = (Absorbance of sample–absorbance of negative control)/ (Absorbance of positive control–absorbance of negative control) × 100(3)

#### 2.2.9. Statistical Analysis

Statistical analysis was performed using GraphPad Prism^®^ software (Version 5.0, San Diego, CA, USA). A non-parametric *t*-test followed by Bonferroni’s multiple comparison post-test was used to compare cellular uptake of nano-formulations.

## 3. Results and Discussion

### 3.1. Determination of Particle Size, PDI, and ZP

**Figure 2:**
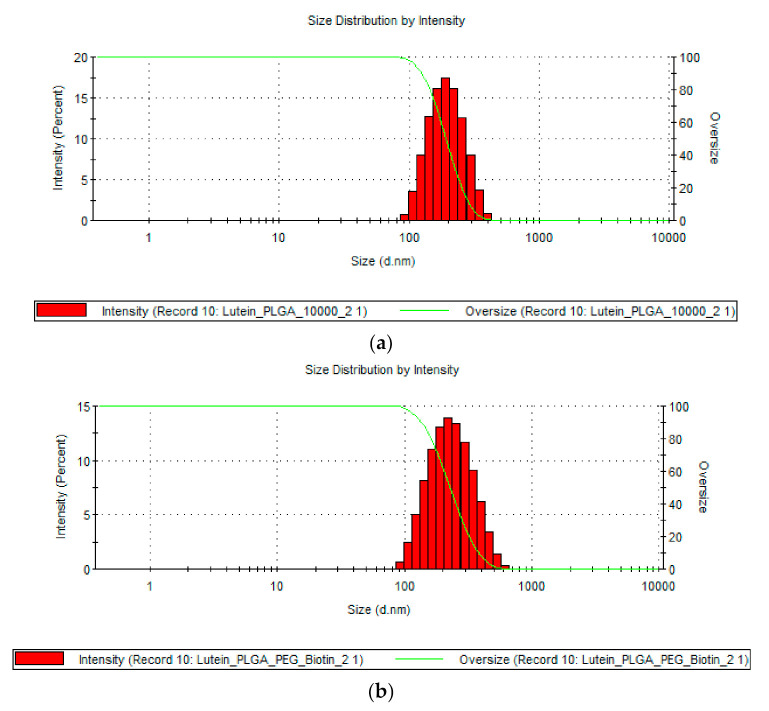
Size distribution curves of (**a**) lutein–PLGA nanoparticles and (**b**) lutein PLGA–PEG–biotin nanoparticles.

The size, PDI, and ZP of lutein-loaded polymeric nanoparticles are provided in Table 1. Results show that all the lutein-loaded nanoparticles had sizes of <250 nm. The sizes of lutein-loaded PLGA and PLGA–PEG–biotin nanoparticles were 196.4 ± 20.04 nm and 208.0 ± 3.38 nm, respectively. Figure 2 confirms the monodisperse distribution of nanoparticles for both formulations. Zeta potential results revealed that lutein-loaded PLGA–PEG–biotin nanoparticles had higher ZP values (−27.2 ± 2.04 mV) compared to lutein-loaded PLGA nanoparticles (−11.2 ± 2.12 mV). The shift of ZP values towards higher negativity could be due to the presence of terminal carboxylic groups in the PEG–biotin portion of block polymer. In addition, negative ZP values result in higher stability of nanoparticles due to prevention of non-specific interaction with proteins in biological proteins. PLGA-based polymers were chosen for this study due to unique properties which include biocompatibility, targetability, biodegradability, and versatile biodegradation kinetics. Peroxisomal degradation of PLGA-based polymers will result in the formation of safe degradation products such as lactic acid and glycolic acid, which are removed by the Kreb’s cycle [48]. However, due to the hydrophobic nature of PLGA, the nanoparticles are rapidly cleared by the mononuclear phagocyte system using opsonization process. Therefore, coating with hydrophilic polymers such as PEG could bypass the opsonization process due to steric repulsion forces. This results in enhancing the bioavailability and half-lives of the drugs by increasing the circulation time of nanoparticles in the plasma [48,49]. Moreover, all the polymers (PLGA, PLGA–PEG–biotin, PVA) are approved by USFDA as inactive ingredients in various formulations [50].

### 3.2. Determination of Encapsulation Efficiency (%EE) and Drug Loading (%DL)

Results showed that PLGA–PEG–biotin polymer had greater lutein encapsulation compared with the PLGA polymer. The entrapment of lutein values were 56.05% ± 7.28% and 74.56% ± 10.25% for lutein PLGA and PLGA–PEG–biotin nanoparticles, respectively. The lutein loading values in PLGA and PLGA–PEG–biotin nanoparticles were ≈11.2% and ≈14.9%, respectively (Table 1).

### 3.3. DSC

**Figure 3:**
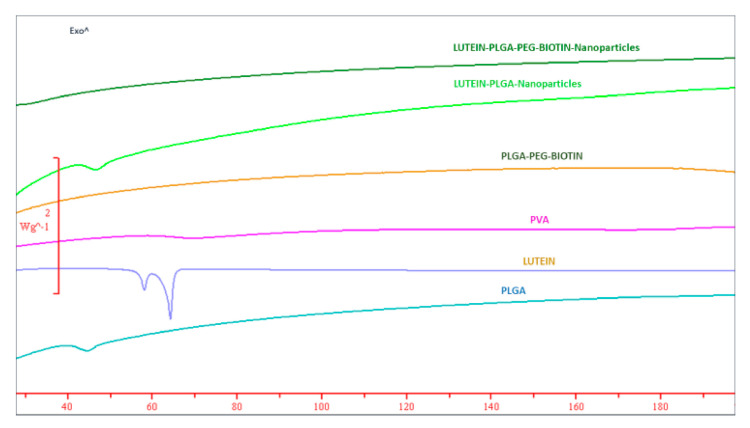
DSC thermograms of lutein-loaded PLGA–PEG–biotin nanoparticles, lutein-loaded PLGA nanoparticles, PLGA–PEG–biotin, PVA, lutein, and PLGA.

DSC is a widely used technique to confirm the polymorphic changes of drugs and polymers in formulation research. Thermograms of lutein, polymers, PVA, and lutein-loaded nanoparticles are provided in Figure 3. Results show that lutein has sharp characteristic endothermic peaks at 64.07 °C and 58.15 °C. In addition, PLGA had a small endothermic peak at 45.28 °C. There was no specific peak/melting point observed for PLGA–PEG–biotin and PVA, suggesting their amorphous nature. The characteristic endothermic peak of lutein disappeared in thermograms of lutein-loaded polymeric nanoparticles, suggesting that lutein was rendered amorphous by its interaction with the polymer. The transformation of lutein from crystalline to amorphous form is important, as amorphous forms are characterized by higher solubilities and increased bioavailability [51,52,53,54]. Similar results (loss of characteristic peak) were observed in other studies where crystalline drugs such as SN-38, doxorubicin oxcarbazepine, prilocaine, and adefovir were encapsulated into PLGA-based micro- and nanoparticles [24,25,55,56,57].

### 3.4. FTIR

**Figure 4:**
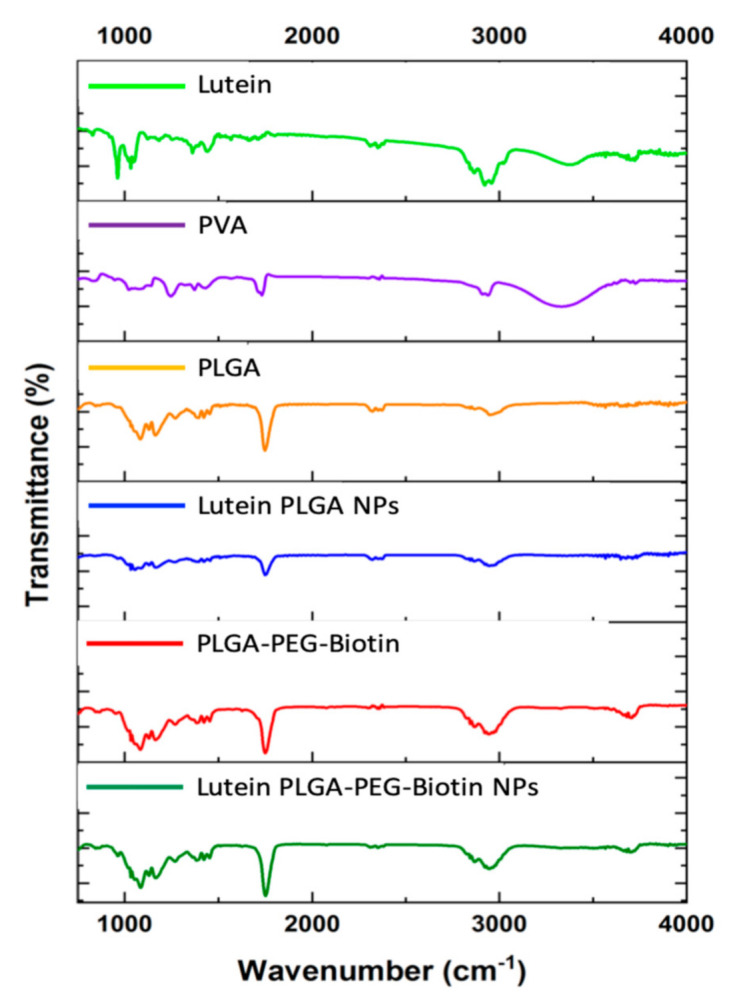
Fourier-transform infrared spectroscopy (FTIR) spectra of freeze-dried lutein-loaded polymeric nanoparticles, PLGA, PLGA–PEG–biotin, PVA, and lutein.

FTIR spectroscopy was used to evaluate the surface chemistries of lutein, PVA, PLGA, PLGA–PEG–biotin, lutein-loaded PLGA, and PLGA–PEG–biotin nanoparticles (Figure 4). The broad transmittance band of lutein between 3588.88 and 3073.01 cm^−1^ corresponds to the OH stretching vibrations; it was absent in lutein-loaded PLGA and PLGA–PEG–biotin nanoparticles. In addition, characteristic C–H stretching bands were observed for lutein at 2958 and 2912 cm^−1^. A strong transmittance of lutein was also observed at 1620.15 cm^−1^ which was absent in the spectra of the lutein-loaded PLGA–PEG–biotin and PLGA nanoparticles. There was no obvious difference in the spectra of the polymers (PLGA and PLGA–PEG–biotin) and lutein-loaded polymeric nanoparticles. Results from FTIR confirm that the characteristic peaks of lutein were absent in the FTIR spectra of lutein-loaded polymeric nanoparticles. This could have been due to the strong interaction of lutein with the polymers which resulted in the absence of characteristic peaks. Similar results were observed for other nanoparticle systems [58]. Moreover, it is evident that the encapsulation of lutein did not result in any structural changes of polymers which might be attributed to either the small concentration of the drug or due to its bonding and non-bonding interactions with the surrounding matrix.

### 3.5. In-Vitro Release Studies

**Figure 5:**
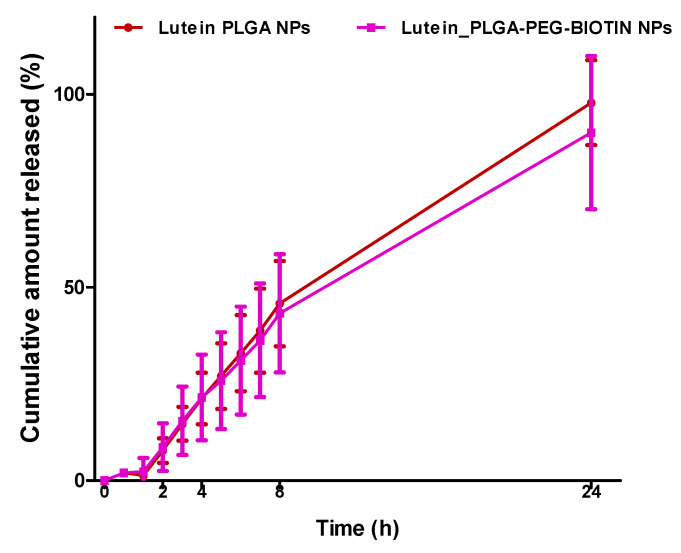
In-vitro release of lutein from lutein-loaded PLGA and PLGA–PEG–biotin NPs. Data are expressed as means ± SDs (n = 3).

The in vitro release profile of lutein from PLGA and PLGA–PEG–biotin nanoparticles is provided in Figure 5. Release profiles revealed that the both the nano-formulations showed similar sustained release patterns with 100% of lutein released within 24 h. Higher encapsulation of lutein in the polymers could have resulted in less space for polymer hydration and led to the slower release of lutein [59]. Overall, release studies validate the sustained/controlled release of drugs from polymeric nanoparticles.

### 3.6. In-Vitro Cellular Uptake Studies

In-vitro cellular uptake of lutein was determined in the ARPE-19 cell line to understand the intracellular localization and uptake of the biotin-decorated PLGA-PEG nanoparticles loaded with lutein compared to lutein–PLGA nanoparticles and lutein alone. These studies also helped to compare the uptake of the lutein nanoparticles within the cytoplasm of ARPE-19 cells. FACS and confocal microscopy were used as quantitative and qualitative analysis methods, respectively, to determine the cellular uptake.

#### 3.6.1. FACS Analysis

**Figure 6:**
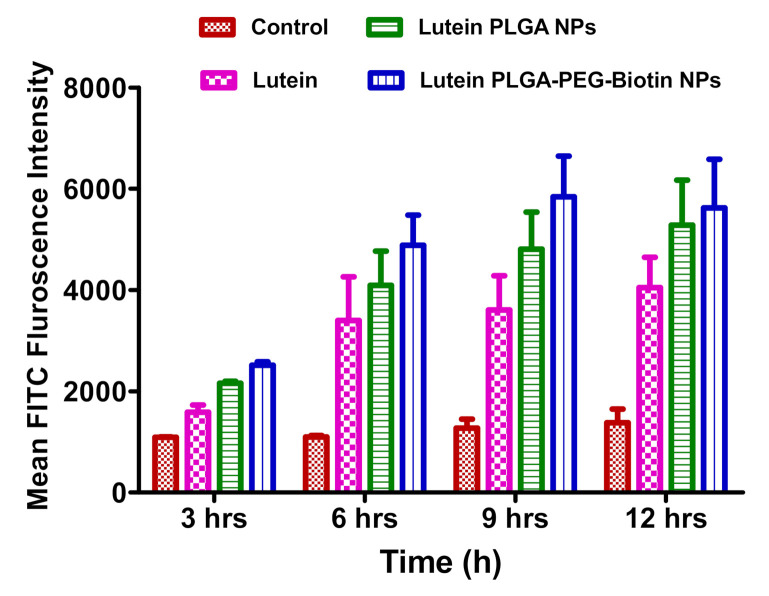
FACS analysis of control, lutein, and lutein-loaded polymeric nanoparticles (PLGA and PLGA–PEG–biotin) for 3, 6, 9, and 12 h in ARPE-19 cells. Data are expressed as means ± SEMs (n = 3).

FITC-labelled, lutein-loaded, biotin-decorated PLGA nanoparticles; PLGA nanoparticles; and lutein alone were incubated with ARPE-19 cells to determine in-vitro uptake at predetermined time points by using flow cytometry (mean FITC florescence intensity). Figure 6 depicts the time- dependent uptakes of FITC-labelled nanoparticles and lutein alone in ARPE-19 cells. Results show that at all time points, there was a significantly higher (*p* < 0.05) mean FITC florescence intensity with lutein-loaded, biotin-decorated nanoparticles and PLGA nanoparticles compared to lutein alone. In addition, it was also observed that at all time points intra cellular uptake of lutein-loaded biotin nanoparticles was numerically higher compared to lutein-loaded PLGA nanoparticles. However, the difference was not statistically significant (*p* > 0.05). Higher uptake with biotin-decorated nanoparticles could be attributed to the presence of a SMVT transporter on the surface of ARPE-19 cells. Results obtained in the study are consistent with other studies evaluating time-dependent uptake of biotinylated nanoparticles for targeted drug delivery [27,56].

#### 3.6.2. Confocal Microscopy

**Figure 7:**
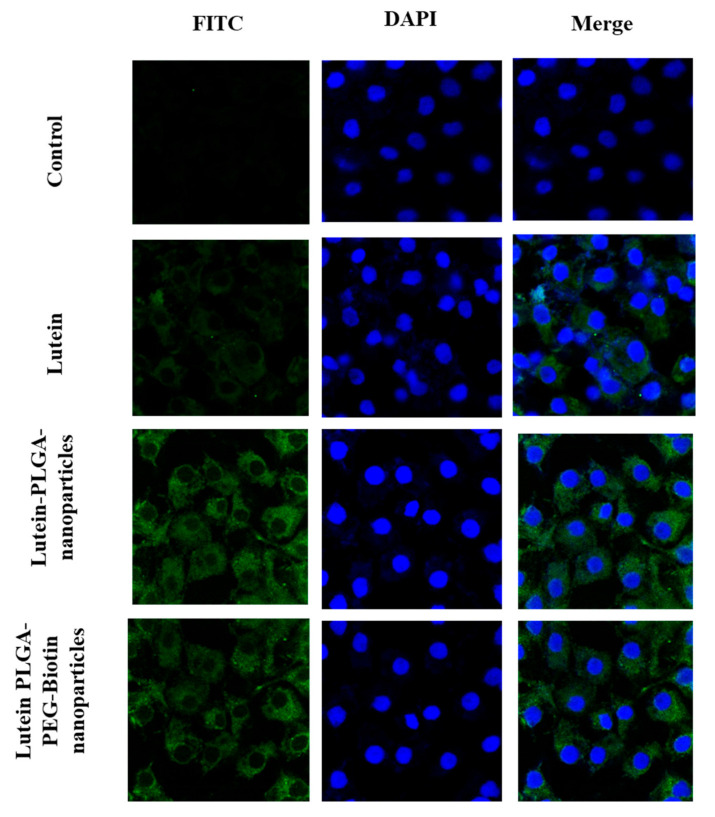
Confocal laser scanning microscopy images of FITC-labelled lutein and FITC-labelled lutein polymeric nanoparticles (PLGA and PLGA–PEG–biotin) at 6 h in ARPE-19 cells.

**Figure 8:**
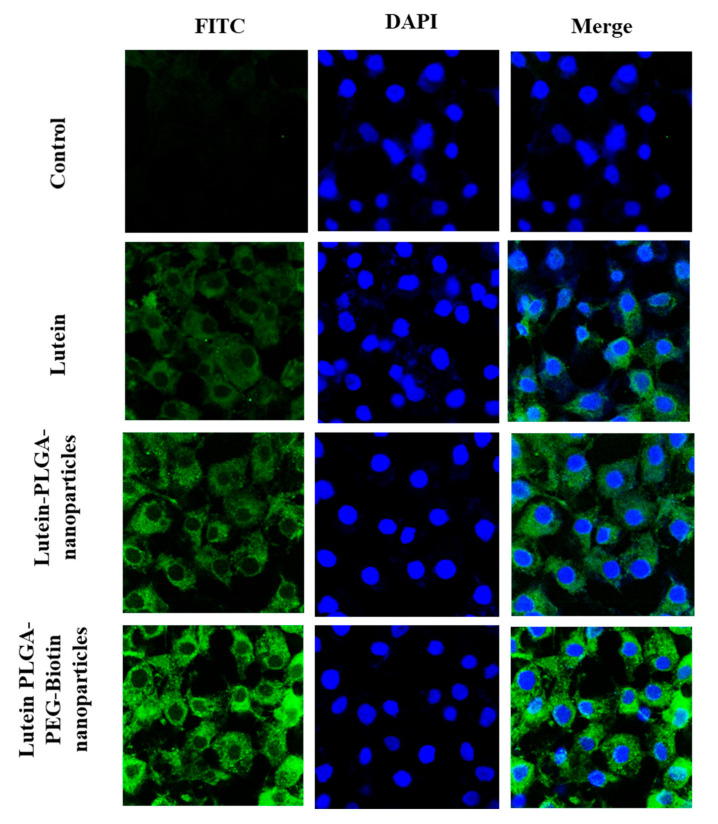
Confocal laser scanning microscopy images of FITC-labelled lutein and FITC-labelled lutein polymeric nanoparticles (PLGA and PLGA–PEG–biotin) at 12 h in ARPE-19 cells.

In-vitro cellular uptake of FITC labelled lutein nanoparticles (PLGA and PLGA–PEG–biotin) and FITC-labelled lutein was performed using confocal laser scanning microscopy for qualitative analysis of lutein uptake by ARPE-19 cells. At the end of each time point the cells were washed, fixed, stained with DAPI, and mounted on a slide for visual observation for confocal microscopy. Figure 7 and Figure 8 depict the in-vitro uptake of FITC-labelled lutein nanoparticles and FITC-labelled lutein at 6 h and 12 h, respectively. It can be observed clearly that the green fluorescence for FITC increased in all the treatment groups with time. It is also interesting to note that at all timepoints, the fluorescence in the cells treated with FITC-labelled, lutein-loaded polymeric nanoparticles (PLGA and PLGA–PEG–biotin) was higher compared to FITC-labelled lutein. These results are well corroborated with the flow cytometry results. This could mean that biotin-decorated lutein nanoparticles and lutein PLGA nanoparticles are well absorbed via the lipid bilayer of ARPE-19 cells as compared to the lutein alone. Thus, the rate of internalization for lutein nanoparticles (PLGA and PLGA–PEG–biotin) into the cytoplasm and nuclei was higher compared to lutein alone. In addition, at 6 and 12 h timepoints, stronger fluorescence was observed with lutein-loaded PLGA–PEG–biotin nanoparticles compared to lutein-loaded PLGA nanoparticles. The higher uptake with biotin-decorated nanoparticles could be attributed to the SMVT transporter mediated uptake in ARPE-19 cells. Higher expression of biotin transporters on the surface of ARPE-19 cells resulted in higher uptake of lutein compared to other treatment groups.

### 3.7. In-Vitro Cytotoxicity Studies

**Figure 9:**
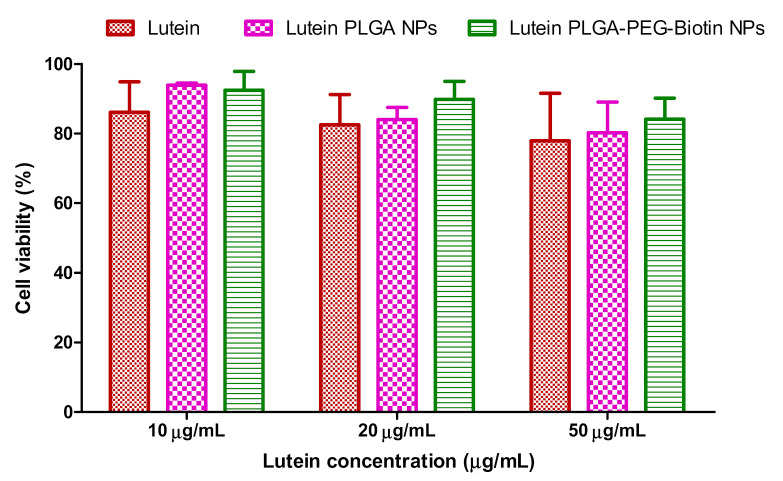
Cell viability (%) of ARPE-19 cells when treated with increasing concentrations of lutein and lutein-loaded polymeric nanoparticles.

Figure 9 shows the cell viability (%) of ARPE-19 cells when treated with increasing concentrations of lutein-loaded polymeric nanoparticles and lutein alone. Lutein and lutein-loaded polymeric nanoparticles did not exhibit any significant cytotoxicity at 10, 20, and 50 µg/mL concentrations, proving that these nano-formulations are safe, effective, and non-toxic. Conjugates of biotin to PLGA nanoparticles did not result in any signs of toxicity and they were equivalent to lutein alone.

## 4. Conclusions

In conclusion, we have successfully developed and characterized lutein-loaded, biotin-decorated polymeric nanoparticles. The obtained nanoparticles possessed particle sizes of <250 nm with narrow size distributions. Moreover, the biotin targeted nanoparticles showed higher encapsulation efficiency and drug loading. In-vitro cellular uptake studies revealed that biotin-decorated nanoparticles exhibited higher uptake of lutein compared to PLGA nanoparticles and lutein alone. In vitro cytotoxicity results confirmed that the nanoparticles showed higher cell viability compared to lutein alone. Collectively, we have found that the biotin-conjugated nanoparticles may be an appropriate formulation for targeted drug delivery in the treatment of AMD and other retinal diseases. However, these results are preliminary and should be confirmed by evaluating the efficacy, safety, and pharmacokinetics in pre-clinical models.

## Figures and Tables

**Table 1:** Size, polydispersity index (PDI), zeta-potential (ZP), and encapsulation efficiency (%EE) of lutein-loaded PLGA and PLGA–PEG–biotin nanoparticles (n = 3). Data are represented as means ± standard deviations (SDs).

S.no	Particle Type	Size (nm)	PDI	ZP (mV)	EE (%)
1	Lutein PLGA	196.4 ± 20.04	0.087 ± 0.016	−11.12 ± 2.12	56.05 ± 7.28
2	Lutein PLGA–PEG–biotin	208.0 ± 3.38	0.206 ± 0.016	−27.2 ± 2.04	74.56 ± 10.25
